# Electrolyte-based calculation of fluid shifts after infusing 0.9% saline in severe hyperglycemia

**DOI:** 10.1186/s40635-020-00345-9

**Published:** 2020-10-13

**Authors:** Robert Svensson, Joachim Zdolsek, Marcus Malm, Robert G. Hahn

**Affiliations:** 1 Department of Anesthesiology and Intensive Care, Vrinnevi Hospital, Norrköping, Sweden; 2 Department of Anesthesiology and Intensive Care, Linköping University, Linköping, Sweden; 3 Department of Biomedical and Clinical Sciences (BKV), Linköping University, Linköping, Sweden; 4 Swedish Defence Research Agency, Linköping, Sweden; 5 Research Unit, Södertälje Hospital, 152 40 Södertälje, Sweden; 6 Karolinska Institutet at Danderyds Hospital (KIDS), Stockholm, Sweden

**Keywords:** Body fluid compartments, analysis, Dehydration, diagnosis, urine, physiopathology, Diabetes mellitus, physiology

## Abstract

**Background:**

Early treatment of severe hyperglycemia involves large shifts of body fluids that entail a risk of hemodynamic instability. We studied the feasibility of applying a new electrolyte equation that estimates the degree of volume depletion and the distribution of infused 0.9% saline in this setting.

**Methods:**

The new equation was applied to plasma and urinary concentrations of sodium and chloride measured before and 30 min after a 30-min infusion of 1 L of 0.9% saline on two consecutive days in 14 patients with severe hyperglycemia (mean age 50 years). The extracellular fluid (ECF) volume was also estimated based on the volume dilution kinetics of chloride.

**Results:**

On day 1, the baseline ECF volume amounted to 11.5 L. The saline infusion expanded the ECF space by 160 mL and the intracellular fluid space by 375 mL. On day 2, the ECF volume was 15.5 L, and twice as much of the infused fluid remained in the ECF space. The chloride dilution kinetics yielded baseline ECF volumes of 11.6 and 15.2 L on day 1 and day 2, respectively. No net uptake of glucose to the cells occurred during the two 1-h measurement periods despite insulin administration in the intervening time period.

**Conclusions:**

The electrolyte equation was feasible to apply in a group of hyperglycemic patients. The ECF space was 3 L smaller than expected on admission but normal on the second day. Almost half of the infused fluid was distributed intracellularly.

## Introduction

High competence is needed to manage fluid, electrolyte, and insulin therapy in patients with poorly controlled diabetes [1]. The severely hyperglycemic patient is always dehydrated, and insulin might aggravate the situation by relocating extracellular fluid (ECF) to the cells, whereby hemodynamic collapse can occur. Adequate matching between fluid and insulin is delicate, and the clinician has to follow early clinical signs to prevent complications [2]. However, few details are known about the fluid shifts that actually occur when treating severely hyperglycemic patients [3].

We have developed an equation system based on a fluid challenge with 0.9% saline followed by measurements of sodium and chloride that estimate the severity of the dehydration and also the fluid-induced shift of ECF volume into the cells.

The aim of the present study was to explore the feasibility of applying the new equation to a group of patients likely to be volume depleted. Severely hyperglycemic patients were considered suitable for this purpose. They receive 0.9% saline and are monitored with measurements of sodium and chloride for clinical reasons.

Our hypothesis was that the calculations would enhance knowledge about the severity of the dehydration and the degree of fluid shifts that occur in the treatment of hyperglycemia. The ECF volume was also calculated with volume kinetic analysis of the plasma electrolyte concentrations over time [4], which is an approach that has been validated by bromide and iohexol dilution [5].

## Methods

An infusion experiment was performed on two consecutive days in 14 fully conscious patients who had been admitted for treatment of poorly controlled diabetes to the intensive care unit (ICU) at the Vrinnevi Hospital in Norrköping, Sweden, between 2014 and 2019. The Regional Ethics Committee of Linköping had approved the protocol (Ref. 2014/123-31), and the study was registered at ClinicalTrials.gov NCT02172092 before any patient was enrolled. Each patient gave his/her oral and written consent for participation.

The patients underwent the first infusion experiment soon after their arrival to the ICU and had the repeat infusion on the next day. Each infusion consisted of 1 L of 0.9% saline over 30 min followed by a free interval of 30 min.

Arterial blood was withdrawn on 9 occasions between 0 and 60 min. The samples were analyzed for plasma concentrations of sodium (Na), chloride (Cl), and ionized calcium (Ca) on a Radiometer ABL 800 FLEX blood gas machine (Radiometer Medical, Copenhagen, Denmark) with a coefficient of variation of approximately 1%.

The patients had a bladder catheter through which the excreted urine was collected, measured, and sampled at 60 min. Urinary electrolytes were measured on the Cobas 8000. The excretion of glucose and electrolytes was taken as the product of the urine volume and the urinary concentration of the solute in question.

Monitoring consisted of pulse oximetry and invasive arterial pressures.

### Electrolyte equation

The background to the proposed electrolyte calculation comes from a previously used mass balance equation based on changes in Na [6, 7]. By assuming that the ECF volume at baseline (time 0) is 20% of the body weight [5], we have only one unknown (∆ICF, flow of fluid to or from the intracellular space) in the following equation, which covers the time period between the baseline and a later time (*t*). In the present study, we use time frame from 0 to 60 min.

$$ \frac{{\mathrm{Na}}_{\mathrm{o}}{\mathrm{E}\mathrm{CF}}_{\mathrm{o}}+\left(\mathrm{infused}-\mathrm{excreted}\right)\mathrm{Na}}{\mathrm{E}{\mathrm{CF}}_{\mathrm{o}}+\left(\mathrm{infused}-\mathrm{excreted}\right)\mathrm{volume}+\Delta \mathrm{ICF}}={\mathrm{Na}}_{\mathrm{t}} $$

All data relating to Na appear in the nominator and the corresponding data for fluid volumes in the denominator. The measured Na_t_ should equal the left side of the equation if ∆ICF is zero. Any deviation in Na_t_ from its theoretical value means that ∆ICF is separated from zero.

Because ECF_o_ cannot be assumed to be 20% of the body weight in poorly controlled diabetes, we strived to develop an equation that determines both ∆ICF and ECF_o_. To solve both unknowns, an equation system was set up that considers the changes in both the Na and Cl concentrations after having infused isotonic saline. Chloride occupies the same physiological space as sodium [8, 9], and the content of both ions in 0.9% saline (154 mmol/L) deviate markedly from the plasma concentrations.

By rearrangement, the equation system holds that the finally arrived Na_t_ and Cl_t_ after infusion of sodium chloride are given by:

$$ \left\{\frac{\frac{{\mathrm{Na}}_{\mathrm{o}}{\mathrm{ECF}}_{\mathrm{o}}+\left(\mathrm{infused}-\mathrm{excreted}\right)\kern0.28em \mathrm{Na}}{{\mathrm{ECF}}_{\mathrm{o}}+\left(\mathrm{infused}-\mathrm{excreted}\right)\kern0.28em \mathrm{fluid}+\Delta \mathrm{ICF}}={\mathrm{Na}}_{\mathrm{t}}}{\frac{\mathrm{Cl}\kern0.5em \mathrm{ECF}+\left(\mathrm{infused}-\mathrm{excreted}\right)\mathrm{Cl}}{{\mathrm{ECF}}_{\mathrm{o}}+\left(\mathrm{infused}-\mathrm{excreted}\right)\mathrm{fluid}+\Delta \mathrm{ICF}}={\mathrm{Cl}}_{\mathrm{t}}}\right) $$

As ∆ICF and ECF_o_ must be the same in both equations, the following re-arrangement can be made and let the electrolyte changes after the fluid challenge estimate ECF_o_:

$$ {\mathrm{ECF}}_{\mathrm{o}}=\frac{\left[\mathrm{N}{\mathrm{a}}_{\mathrm{t}}\kern0.5em \left(\mathrm{infused}-\mathrm{excreted}\right)\mathrm{Cl}\right]-\left[{\mathrm{Cl}}_{\mathrm{t}}\kern0.5em \left(\mathrm{infused}-\mathrm{excreted}\right)\mathrm{Na}\right]}{\left({\mathrm{Na}}_{\mathrm{o}}\kern0.5em {\mathrm{Cl}}_{\mathrm{t}}\right)-\kern0.5em \left(\mathrm{N}{\mathrm{a}}_{\mathrm{t}}\kern0.5em {\mathrm{Cl}}_{\mathrm{o}}\right)} $$

In turn, ∆ICF that has occurred from baseline to time (*t*) can be solved as:

$$ \Delta \mathrm{ICF}=\frac{\left[{\mathrm{N}\mathrm{a}}_{\mathrm{o}}{\mathrm{ECF}}_{\mathrm{o}}+\left(\mathrm{infused}-\mathrm{excreted}\right)\mathrm{Na}\right]}{\mathrm{N}{\mathrm{a}}_{\mathrm{t}}}-{\mathrm{ECF}}_{\mathrm{o}}-\left(\mathrm{infused}-\mathrm{excreted}\right)\mathrm{fluid} $$

The change in ECF volume up to the later time *t* can now be obtained as

$$ \Delta  \mathrm{ECF}=\left(\mathrm{infused}-\mathrm{excreted}\right)\ \mathrm{fluid}-\Delta  \mathrm{ICF} $$

### Volume kinetic analysis

In the present study, a one-compartment volume kinetic analysis was applied to the infused fluid load, using the Cl concentration measured at 0, 10, 20, 30, 35, 40, 45, 50, and 60 min as index of dilution. However, the dilution of Cl could not be applied directly as a considerable amount of Cl was infused. Therefore, the kinetic analysis was based on the volume of distribution of Cl, denoted as *v*, which constantly changes as fluid is infused and eliminated. The key output measure is still ECF_o_.

**Fig. 1:**
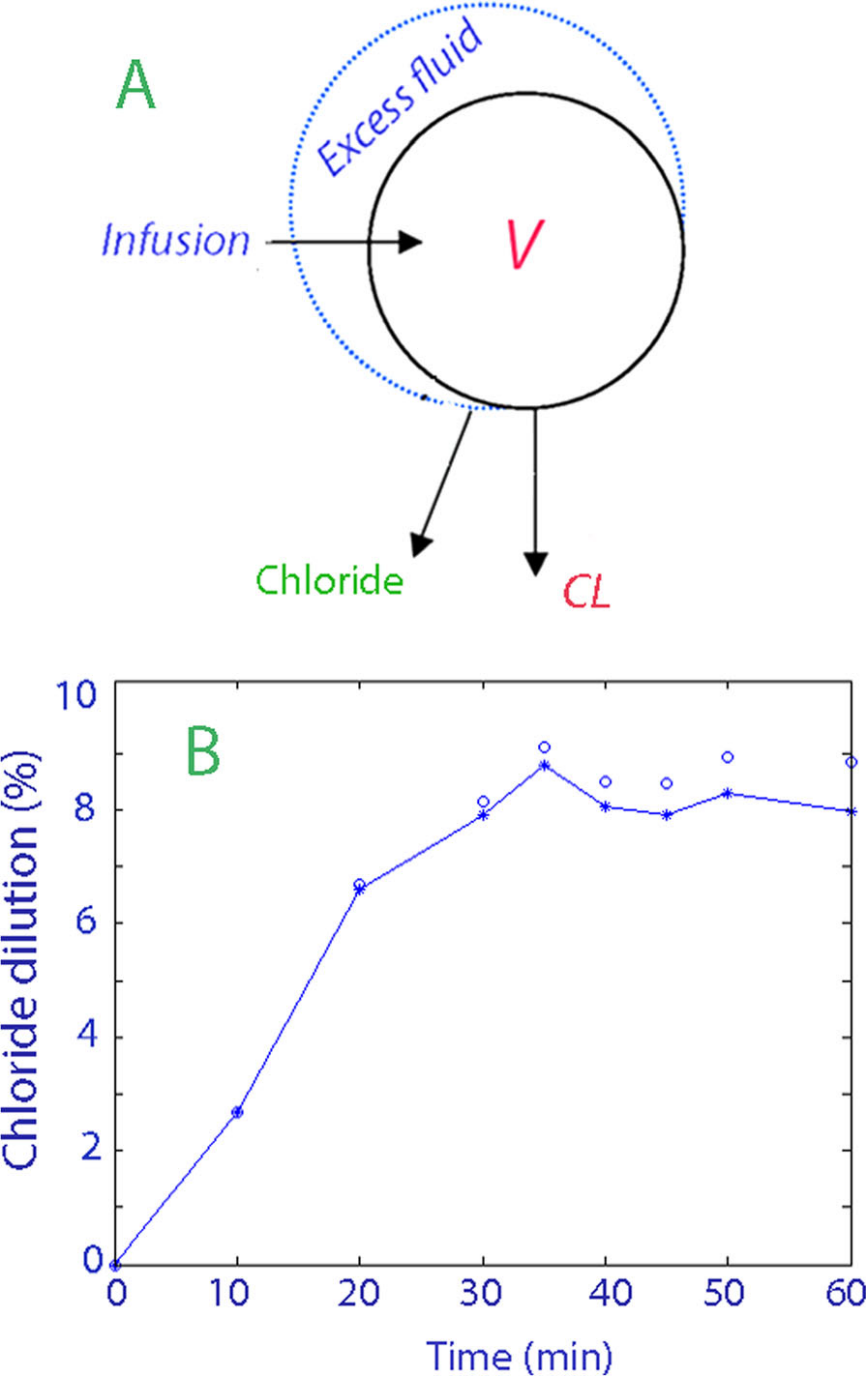
**a** Schematic drawing of the model used for the analysis of chloride dilution kinetics. CL is the renal fluid clearance and “Chloride” represents the urinary excretion of chloride ions. **b** Chloride dilution of the extracellular fluid space (ECF). Open circles are the measured chloride dilutions corrected for the addition of Cl with the 0.9% saline and the connected filled circles the dilution after correction also for urinary losses of chloride

In the kinetic model, fluid is infused in ECF_o_, which is then expanded to (*v* − ECF_o_) (Fig. 1a). Elimination occurs at a rate proportional to the dilution of the ECF volume, i.e., (*v* − ECF_o_)*/*ECF_o_ (dependent variable), by the renal fluid clearance, CL (as 0.9% saline is eliminated via the kidneys). The change of the ECF volume is then described by the following differential equation [4, 5]:

$$ \mathrm{d}v/\mathrm{dt}=\mathrm{Infusion}\ \mathrm{rate}-\mathrm{CL}\left(v-{\mathrm{ECF}}_{\mathrm{o}}\right)/{\mathrm{ECF}}_{\mathrm{o}} $$

Correction for losses and additions of Cl was done by taking the total amount of chloride in the ECF volume as the product of ECF_o_ (as given by the electrolyte equation) and Cl_o_ to which the infused amount of chloride was added. Losses of chloride were taken as the total excreted amount at 60 min divided by the area under the curve (AUC) for the dilution of plasma Cl but considering only a fraction of AUC for the time segment up to each time *t* [4]. Hence, the volume of distribution of Cl, which is chloride amount/Cl_t_ and represented by the symbol *v*, was given by:

$$ \left[\ {\mathrm{ECF}}_{\mathrm{o}}\ {\mathrm{Cl}}_{\mathrm{o}}+\mathrm{infused}\ \mathrm{Cl}-\left[\frac{\mathrm{excreted}\ \mathrm{Cl}\ \mathrm{at}\ 60\ \min }{\mathrm{AUC}\ \mathrm{for}\ \mathrm{dilution}\ \mathrm{of}\ \mathrm{plasma}\ \mathrm{Cl}}\ \left(\mathrm{t}-{\mathrm{t}}_{\mathrm{o}}\right)\ {\mathrm{Cl}}_{\mathrm{t}}\ \right]\ \right]/{\mathrm{Cl}}_{\mathrm{t}} $$

where each of the 9 time segments are denoted (*t* − *t*_o_). ECF_o_ was the key outcome measure of the subsequent volume kinetic calculation. The kinetic calculations were performed by least-squares regression based on a Gauss-Newton routine, using Matlab version 4.2 (Math Works Inc., Notich, Mass) [4].

An exploratory analysis of the dilution of plasma Ca was also performed. The dependent variable was then (Ca_o_ − Ca_t_)/Ca_t_ (here, it is important that the diluted concentration is placed in the denominator). A non-compartment model analysis (NCA) was chosen because the maximum dilution occurred with a delay of 10–15 min from the end of the infusion. The Phoenix 8.2 software (Pharsight, St. Louis, MO) was used for the analysis, which was designed as “exploratory” because urinary losses for Ca were not measured. However, these losses are small in response to infusion of 0.9% saline [10, 11], and they primarily affect the clearance of the infused fluid volume.

A kinetic analysis of the blood hemoglobin data from these patients has been published previously [12].

### Statistics

Group data are presented as the mean and deviation (SD) and the paired *t* test was used for selected statistical comparisons of changes during experiments. Only mean values were applied to the equations because calculations based on individuals would be too much affected by the precision for the electrolyte measurements. Calculations were conducted using the StatView SE+Graphics v.1.02 software (Abacus Concepts, NJ), and *P* < 0.05 accepted as statistically significant.

The sample size was not determined by a power calculation as the purpose of the study is the feasibility of estimating the body fluid volumes from changes in electrolytes after infusing 0.9% saline.

## Results

The 14 patients were aged between 18 and 86 (mean, 50) years and had body weight of 71 (SD, 11) kg. Eight patients had been given 1 L of buffered Ringer’s solution before admission to the ICU, and 3 had received 6–10 units of insulin. The second infusion took place 20.7 (4.2) h after the first one. The patients had then received a total of 7.0 (1.9) L of crystalloid fluid and 26 (16) units of insulin since their arrival to the ICU.

### Blood and urine chemistry

Blood and urine chemistry and hemodynamic data are shown in Table 1. Before the first infusion, the patients had severe hyperglycemia (mean 35.4 mmol/L). The first infusion reduced plasma lactate concentration (*P* < 0.02) but had little acute effect on the systemic acidosis. Plasma glucose decreased by 20% (*P* < 0.001). There was profuse glucosuria that averaged to almost 100 mmol during the hour of study in response to the saline. The glucosuria was only 1/4 as large during the second infusion, when many of the blood chemistry parameters had been restored to be within the normal range.

**Table 1:** Fluid balance variables

	Day 1		Day 2	
	0 min	60 min	0 min	60 min
Blood chemistry				
P-glucose (mmol/L)	35.4 (9.4)	29.8 (8.3)	14.5 (2.9)	14.1 (3.3)
P-Na (mmol/L)	132.3 (6.1)	132.8 (5.3)	137.7 (6.0)	136.1 (5.3)
P-Cl (mmol/L)	92.8 (6.3)	97.4 (6.7)	104.9 (7.4)	105.3 (5.9)
P-Ca ionized (mmol/L)	1.33 (0.09)	1.26 (0.07)	1.24 (0.06)	1.19 (0.05)
P-Mg (mmol/L)	0.90 (0.23)	0.89 (0.17)	0.82 (0.13)	0.77 (0.14)
B-Hb (g/L)	147.8 (26.9)	139.8 (22.9)	124.2 (21.1)	119.6 (17.6)
P-lactate (mmol/L)	1.72 (0.63)	1.17 (0.37)	0.94 (0.51)	0.92 (1.10)
pH	7.24 (0.09)	7.23 (0.09)	7.40 (0.07)	7.38 (0.07)
Base excess (mmol/L)	− 15.2 (7.1)	− 15.6 (6.9)	− 4.2 (5.5)	− 5.2 (5.8)
Urine analyses				
U-volume (mL)		468 (344)		202 (127)
U-glucose (mmol/L)		189.5 (69.0)		113.4 (102.9)
U-Na (mmol/L)		47.6 (25.0)		75.9 (38.0)
U-Cl (mmol/L)		23.9 (14.2)		105.4 (44.4)
U-Mg (mmol/L)		1.20 (0.83)		2.95 (1.14)
Hemodynamics				
Arterial pressures				
Systolic (mmHg)	123.9 (29.1)	126.4 (23.5)	139.2 (26.1)	136.0 (24.1)
Diastolic (mmHg)	64.3 (12.5)	60.9 (16.5)	62.2 (10.9)	63.5 (17.2)
Heart rate (bpm)	104.2 (22.0)	99.6 (19.9)	86.1 (12.3)	86.2 (10.3)

Concentrations are given in mmol/L (except where noted) and volumes (total) in L. Data are the mean (SD)

### Electrolyte equation

The electrolyte equation was applied for the entire period between 0 and 60 min. The calculation employed the mean plasma concentrations of Na and Cl at 0 and 60 min and the mean excreted fluid volume and amounts of Na and Cl at 60 min.

On day 1, the ECF volume before the first infusion was initiated amounted to 11.5 L, the expansion of the ECF space from the infusion was 160 mL, and the expansion of the ICF was 375 mL. The remainder of the 1 L of infused fluid had been excreted as urine.

On day 2, the ECF volume before the infusion started was 15.5 L, the expansion of the ECF volume was 377 mL, and the expansion of the ICF was 385 mL.

### Volume kinetic analyses

The ECF volume was estimated by volume kinetic analysis based on the dilution of the mean plasma chloride concentration for the 14 patients on the 9 points of measurement. A schematic drawing of the kinetic model is shown in Fig. 1a and the effect of the correction of the dilution for chloride excretion is illustrated in Fig. 1b.

This analysis based on Cl showed that the size of ECF_o_ on day 1 was 11.6 (0.8) L. On day 2, the size of *V* amounted to 15.2 (1.1) L.

The exploratory analysis based on Ca showed that the ECF_o_ at steady state on day 1 was 11.7 L. On day 2, the size of *V* amounted to 14.8 L.

### Integrated view

**Fig. 2:**
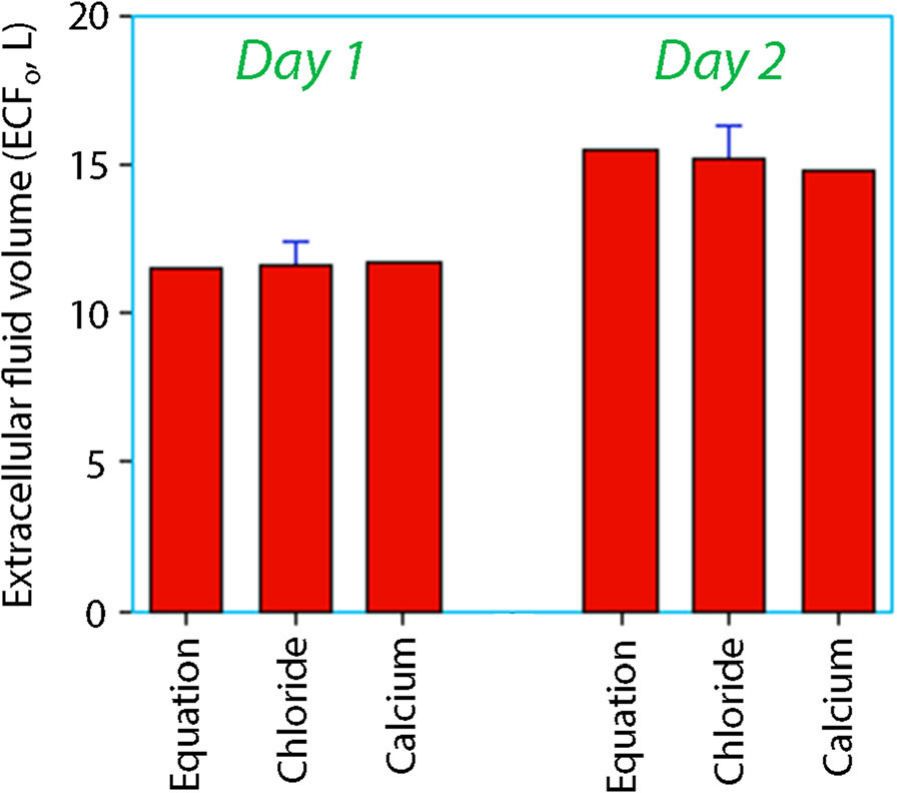
Estimates of the extracellular fluid volume (ECF) on day 1 and day 2 obtained by the electrolyte equation and by kinetic analysis of the dilution data on chloride and calcium

**Fig. 3:**
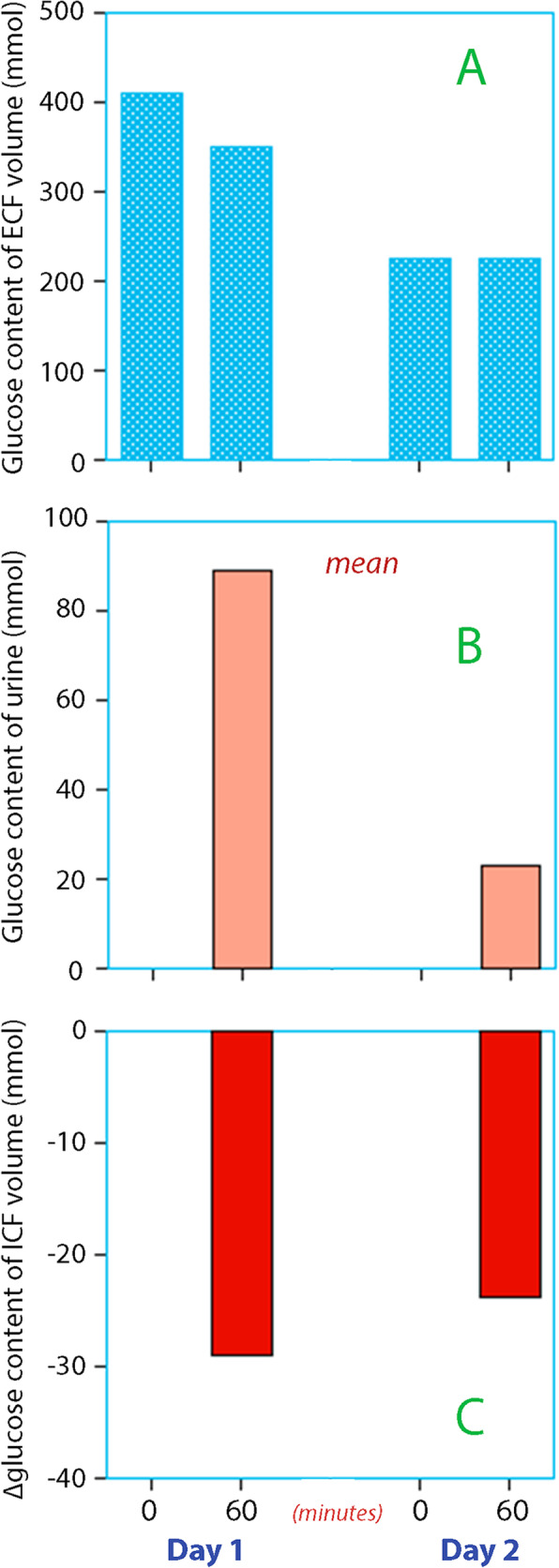
**a** Glucose content of the extracellular fluid before and after infusion of 0.9% saline on two consecutive days. The data are the product of plasma glucose and the estimates of extracellular fluid volume as obtained by the new electrolyte equation. **b** Excreted amount of glucose during the 60-min infusion experiment. **c** Net release of glucose from the body cells during the 60-min experiment needed to account for the excreted glucose and the amount remaining in the extracellular fluid

Figure 2 highlights the estimates of the ECF volume, and Fig. 3 gives an integrated view of the amounts of glucose in the ECF and urine volumes, and also the flux of glucose to or from the ICF (= the difference between the other two variables). Figure 3 shows that glucose content of the ECF was reduced by 50% between day 1 and day 2, and the glycosuria dropped by 75%. However, the output of glucose from the cells remained at 25–30 mmol/h despite the administration of insulin.

## Discussion

This study comprises patients who had been admitted to ICU for treatment of poorly controlled diabetes, most of which had developed acidosis. Only few had received modest initial treatment with fluid and insulin before being enrolled in the study. We applied a new mass balance approach based on electrolyte shifts to estimate the distribution of short-term infusions of crystalloid fluid on two consecutive days.

The results show that the ECF space had a volume of approximately 11.5 L on admission, which is almost 3 L less than expected based on tracer measurements in healthy individuals [5]. The total fluid deficit would certainly be much greater if the ICF had been included in this figure.

Most of the subsequently infused fluid distributed equally between the urine and the ICF space, whereas only 1/6 hydrated the ECF. On day 2, the deficit in the ECF had been corrected, and perhaps even slightly overcompensated. The electrolyte equation further implicated, in light of a smaller diuretic response to the saline, that the infused volume now distributed equally between the ECF and the ICF.

Isotonic (0.9%) saline is a fluid composed to remain only the ECF volume, and the pronounced distribution to the ICF on day 1 may then seem odd. Prior to this study, we believed that administration of insulin would promote relocation of fluid to the ICF. However, not even on day 2 had the insulin reversed the catabolic state and promoted uptake of glucose to the ICF volume. This finding might be due to insulin resistance being maintained due to the hyperglycemic environment “glucose toxicity” [13].

The intracellular distribution of infused fluid seemed to be better explained by the glycosuria. When diabetic hyperglycemia develops, gluconeogenesis and glycogenolysis release glucose that translocates ICF water to the ECF by virtue of osmosis. When this glucose is excreted, the osmotic “power” to hold the translocated fluid in the ECV is lost, and it returns to the cells. The lack of glucose uptake also suggests that glycosuria was the key factor, and perhaps the only one, responsible for the decrease in plasma glucose during the first 24 h of treatment.

Intravenous fluid should be the initial treatment of poorly controlled diabetes, and the fluid shifts reported in the present study highlight why they entail a risk of inducing hemodynamic instability [1, 2]. The effectiveness of insulin seems to be poor and, therefore, the effect of early insulin treatment at this stage should mainly be to alleviate the acidosis. The recommended administration of insulin is 0.1 units/kg/h [14]. The risk of hypovolemic hypotension would probably be much greater if pronounced if early uptake of glucose to the ICF occurred.

Bergamasco et al. [15] have developed equations to help estimate fluid volume derangements by comparing measured with estimated normal values of plasma sodium, plasma glucose, and serum osmolality. Olde Engeberink et al. compared several similar equations with new experimental data [16]. All these equations make assumptions about the size of body fluid volumes at baseline, which is problematic in the presence of severe fluid derangements. Alternatively, baseline volumes can be measured by radioactive tracer technologies, but these are hardly ethical to apply in acute clinical situations.

Our current approach overcomes these shortcomings by analyzing the electrolyte changes induced by a fluid challenge. No radioactive tracer is needed and no assumptions about body fluid volumes or “normal” electrolyte concentrations have to be made. Only two plasma samples and one urinary sample are required for the calculations, and only two electrolyte concentrations need to be analyzed. The measurements, as well as the fluid load, are likely to be implemented for clinical purposes anyway, which makes ethical aspects a less crucial issue.

The new electrolyte equation was derived from an existing equation used to estimate ICF distribution of hypo-osmotic irrigating fluid in patients with an assumed normal ECF space [6, 7]. The change in plasma sodium resulting from dilution with sodium-free fluid can be predicted by assuming a uniform distribution of sodium (not fluid) within the ECF volume. Any deviation from the predicted change implies that fluid has passed across the cell membrane. However, in poorly controlled diabetes, one cannot assume that ECF is normal. Therefore, we combined the sodium equation with a similar equation using chloride shifts, which allowed the size of ECF to be estimated because both ions should indicate the same flow of fluid across the cell membrane.

The new equation is mixture of dilution and electrolyte kinetics and provides information about both the degree of pre-infusion volume depletion and the subsequent fluid shift between the ECF and the ICF during the measurement period. The first parameter, but not the second, can also be obtained by applying volume kinetic analysis to a serial analysis of extracellular electrolytes [4, 5].

We used chloride for such a complementary calculation, and estimates of ECF_o_ were quite similar to those found by the electrolyte equation. However, the kinetic analysis of Cl may not be considered a control method because some data were used in both approaches, although the kinetic analyses used many more of the performed measurements. By contrast, the volume kinetic analysis based on plasma Ca was completely independent of the electrolyte equation, as 0.9% saline solution does not contain calcium. Unfortunately, the Ca dilution showed a delayed time course that necessitated the use of an alternative kinetic model not planned for when the study started.

Limitations include that only 1 L of saline was infused, and the changes in the plasma concentrations of sodium and chloride were not large enough to safely overcome the associated measurement errors in individual patients. With the present set-up, we recommend that the equation is applied only to group data. The electrolyte changes should be greater to allow reasonably safe application of the new equation on individual patients. Hence, the electrolyte equation was probably more accurate on day 1 than on day 2 because the 0.9% saline induced greater changes in plasma electrolytes at that time. Infusing a larger volume of saline, 2 L rather than 1 L, might overcome the confounding effect of errors in sampling and analysis of electrolyte concentrations.

The electrolyte equation does not take non-osmotic sodium, which is found in the bone, skin, and in the glycocalyx, into account. Olde Engeberink et al. showed that a significant amount of sodium was stored in the body when hypertonic saline was infused rapidly to sodium-depleted volunteers [16]. However, the surplus of sodium in the 0.9% saline we infused is limited. Furthermore, the glycocalyx is damaged in diabetic hyperglycemia [17] and, therefore, might lack adequate storage capacity [18].

Limitations also include that plasma sodium but not the plasma chloride is known to have a somewhat lower concentration in the interstitial fluid than in the plasma because most plasma proteins have negative charge (the Donnan effect). Experiments with radioactive Na and Cl show a 3% smaller ECF_o_ for Cl than for Na, although these calculations were not corrected for urinary losses of tracer electrolytes [9]. The relevance of the above concerns is unclear at this time, but the body volumes obtained by the electrolyte equation might be regarded as functional volumes until validated with isotope dilution. Nevertheless, kinetic analysis based on sodium dilution during infusion of isotonic (5%) mannitol has previously showed excellent correlation with bromide and iohexol measurements of the ECF volume in volunteers [5].

In conclusion, a new mass balance equation based on plasma and urinary electrolytes as well as fluid volume kinetics based on chloride dilutions showed an extracellular fluid deficit of approximately 3 L in patients with poorly controlled diabetes admitted to the ICU. The first liter of 0.9% was distributed mainly to the ICF and the urine. Hydration of the ECF improved when the infusion was repeated on the next day. The decrease in plasma glucose during the first hour of fluid treatment was due to osmotic diuresis.

## Data Availability

All data are available on request to the corresponding author.
